# Re-incubation of positive anaerobic blood culture bottles for detecting additional anaerobes in polymicrobial bacteremia

**DOI:** 10.1128/spectrum.01551-26

**Published:** 2026-08-03

**Authors:** Kensuke Shimbara, Hiroki Kitagawa, Kayoko Tadera, Yuta Kuhara, Keitaro Omori, Norifumi Shigemoto, Shinya Takahashi, Hiroki Ohge

**Affiliations:** 1 Department of Surgery, Graduate School of Biomedical and Health Sciences, Hiroshima University 592177, Hiroshima, Japan; 2 Department of Infectious Diseases, Hiroshima University Hospital 68272, Hiroshima, Japan; 3 Section of Clinical Laboratory, Division of Clinical Support, Hiroshima University Hospital 68272, Hiroshima, Japan; 4 Division of Laboratory Medicine, Hiroshima University Hospital 68272, Hiroshima, Japan; 5 Translational Research Center, Hiroshima University 592315, Hiroshima, Japan; Ascension St John Hospital, Detroit, Michigan, USA

**Keywords:** anaerobic bacteremia, obligate anaerobes, polymicrobial bacteremia, blood culture, MALDI-TOF mass spectrometry, diagnostic microbiology

## Abstract

Anaerobes are important causes of bloodstream infections, but may be overlooked in routine blood culture workflows because of their slow growth and frequent involvement in polymicrobial infections. We hypothesized that in polymicrobial bacteremia, rapidly growing organisms may trigger early blood culture positivity before slow-growing anaerobes have sufficiently proliferated, potentially leading to underdetection during routine subculture. In this observational study, we evaluated whether re-incubation of positive anaerobic blood culture bottles improves the detection of anaerobic bacteria. All blood samples were cultured using the BacT/Alert Virtuo automated blood culture system. Adult patients with at least one positive anaerobic blood culture bottle were included. Positive anaerobic bottles were subcultured using the conventional method and then re-incubated for an additional 4 days before repeat subculture to detect additional anaerobes. Species were identified using matrix-assisted laser desorption/ionization time-of-flight mass spectrometry, supplemented by 16S rRNA gene sequencing. Of 310 patients with positive anaerobic blood cultures, 29 (9.4%) had an additional 36 bacterial isolates identified after re-incubation, of which only 3 were judged to be contaminants. Of 27 cases with two positive anaerobic bottles, additional organisms were detected in both bottles in 23 cases (85%), and concordant organisms were detected from the suspected infection focus in 9 cases (31%). Additional species identified following repeat incubation included *Bacteroides fragilis*, *Veillonella parvula*, *Parvimonas micra*, *Dysgonomonas capnocytophagoides*, and *Eggerthella lenta*. This re-incubation approach provides microbiological evidence that slow-growing anaerobes may be underdetected in routine blood culture workflows for polymicrobial bloodstream infections.

**IMPORTANCE:**

Anaerobes are important causes of bloodstream infections but may be overlooked in routine blood culture workflows. In polymicrobial infections, rapidly growing microorganisms may trigger early blood culture positivity before slow-growing anaerobes have had adequate time to proliferate, leading to missed detection of slower-growing anaerobes during routine subculture. In this study, we re-incubated positive anaerobic blood culture bottles for an additional 4 days after they became positive to provide more time for slower-growing anaerobes to grow. This led to the detection of 36 additional bacterial isolates that would otherwise have remained undetected, of which only 3 were judged to be contaminants. Some of these slow-growing microorganisms are rarely reported in bloodstream infections and some were concordant with anaerobic microorganisms isolated from the suspected infection focus. These findings provide microbiological evidence that slow-growing anaerobes may be underdetected in routine workflows for polymicrobial bloodstream infections, although prolonged turnaround time limits routine implementation.

## INTRODUCTION

Anaerobic bacteremia accounts for approximately 0.5%–9% of all bloodstream infections detected in blood cultures (1–4). The most common source of anaerobic bacteremia is intra-abdominal infections, followed by oropharyngeal, female genital tract, and soft tissue infections (1–6). The most frequently detected anaerobic bacteria in bacteremia include *Bacteroides*, *Clostridium*, and *Fusobacterium* species (1–6). Anaerobic bacteria generally grow more slowly than aerobic bacteria, and therefore the time to positivity (TTP) in automated blood culture systems is often longer (7, 8). In addition, approximately 30% of cases of anaerobic bacteremia are polymicrobial (9, 10).

We have previously reported cases of bacteremia caused by obligate anaerobes, of which many required more than 2 days to become positive on blood culture (11–15). Based on these observations, we hypothesized that in polymicrobial bacteremia involving anaerobic bacteria, rapidly growing organisms, such as Enterobacterales, may trigger early blood culture positivity and that if anaerobic subculture is performed at that time, slower-growing anaerobes may not have proliferated sufficiently and may therefore remain undetected in subculture performed according to routine methods.

Previous studies conducted in the 1980s found that re-incubation of positive blood culture bottles and additional subcultures rarely identified polymicrobial bacteremia and provided limited clinical benefit in automated blood culture systems (16, 17). However, modern automated systems, such as the BacT/Alert Virtuo (bioMérieux, Marcy l’Étoile, France), have reduced the TTP of blood cultures for most bloodstream pathogens (18–21), potentially leading to earlier bottle positivity in polymicrobial infections. In contrast, for obligate anaerobes, particularly *Bacteroides* species, the Virtuo system may detect growth several hours later than the BacT/Alert 3D system (bioMérieux) (22). Therefore, this study aimed to determine whether re-incubation of positive anaerobic blood culture bottles could detect additional anaerobes that were not recovered by the conventional subculture method using the BacT/ALERT Virtuo system.

## RESULTS

The analysis included 531 anaerobic blood culture bottles from 310 patients. Among the 310 patients, 29 (9.4%) had an additional 36 bacterial isolates isolated after re-incubation. The clinical characteristics of patients in the standard-method-only and re-incubation-positive groups are summarized in Table 1. Intra-abdominal infection was the most common source of bacteremia in both groups. Oropharyngeal infections were significantly more frequent in the re-incubation-positive group (*P* = 0.030).

**TABLE 1:** Clinical characteristics of patients in this study^*a*^

Variable	All patients (*n* = 310)	Standard-method-only group (*n* = 281)	Re-incubation-positive group (*n* = 29)	*P* value
Age (years), median (IQR)	74 (61–80)	73 (61–80)	76 (61–84)	0.24
Male, *n* (%)	194 (62.6)	176 (62.6)	18 (62.1)	>0.999
Source of bacteremia, *n* (%)				
Intra-abdominal	165 (53.2)	148 (52.7)	17 (58.6)	0.56
Liver or biliary tract	129 (41.6)	120 (42.7)	9 (31.0)	0.22
Intestinal perforation	16 (5.2)	14 (5.0)	2 (6.9)	0.65
Urinary tract	53 (17.1)	51 (18.1)	2 (6.9)	0.19
Vascular catheter	16 (5.2)	16 (5.7)	0 (0)	0.38
Skin and soft tissue	14 (4.5)	11 (3.9)	3 (10.3)	0.13
Respiratory	16 (5.2)	15 (5.3)	1 (3.4)	>0.999
Oropharynx	8 (2.6)	5 (1.8)	3 (10.3)	0.030
Female genital tract	3 (1.0)	2 (0.7)	1 (3.4)	0.26
Others	35 (11.3)	33 (11.7)	2 (6.9)	0.77
Blood culture				
No. of positive anaerobic bottles, *n*	531	475	56	
Time to positivity (h), median (IQR)	11.2 (9.1–17.6)	11.1 (9.1–16.8)	11.4 (8.9–23.6)	0.49

^*a*^ IQR, interquartile range. The number of positive anaerobic bottles represents the total number of positive bottles rather than a patient-level variable; therefore, no statistical comparison was performed.

The clinical characteristics of the patients and the anaerobic bacteria detected in the re-incubation-positive group are summarized in Table 2. Gram-negative bacilli were the predominant additional anaerobic organisms detected by the re-incubation method, followed by Gram-positive non-spore-forming bacilli (Table 3). *Bacteroides fragilis* and *Veillonella parvula* were the most frequently detected organisms (three cases each), followed by several species including *Parvimonas micra*, *Dysgonomonas capnocytophagoides*, and *Eggerthella lenta* (two cases each). Among the 27 cases with two positive anaerobic bottles, additional organisms detected by the re-incubation method were identified in both bottles in 23 cases (85%), and in 9 cases (31%), these organisms were also isolated from specimens obtained from the suspected infection focus. Two cases with *Cutibacterium acnes* isolation and one case with *Staphylococcus epidermidis* isolation were considered contaminants. In Patient 16, *E. lenta* was identified by the re-incubation method from a bottle with a TTP of 22.1 h, whereas it was later identified by the standard method from an anaerobic bottle with a TTP of 43.9 h. In patient 18, one anaerobic bottle became positive at 18.7 h and yielded only *Streptococcus anginosus* by the standard method, whereas *Desulfovibrio fairfieldensis* was identified by the re-incubation method. The second anaerobic bottle became positive at 94.9 h and yielded *D. fairfieldensis* by the standard method, resulting in its detection in both anaerobic bottles.

**TABLE 2:** Clinical and microbiological characteristics of patients in the re-incubation-positive group^*f*^

Pt. no.	Age/sex	Infection source (source category)	TTP of anaerobic bottles (h)	Organisms detected by the standard method	Additional organisms detected by the re-incubation method	Contamination	Detected in both anaerobic bottles	Concordance with isolates from the infection focus
1	53/M	Dental abscesses (oropharynx)	23.8, 23.8	*Streptococcus constellatus*, *Parvimonas micra*	*Dialister pneumosintes*	No	Yes	No
2	49/M	Aorto-esophageal fistula with mediastinitis (others)	17.6, 18.4	*Neisseria subflava*, *Granulicatella adiacens*, *Fusobacterium nucleatum*	*Campylobacter concisus*	No	Yes	No
3	91/M	Odontogenic maxillary osteomyelitis (oropharynx)	11.5, 11.5	*Streptococcus constellatus*	*Parvimonas micra*	No	Yes	Yes
4	61/M	Cholangitis (intra-abdominal)	9.1, 9.6	*Escherichia coli*, *Staphylococcus aureus*	*Dysgonomonas capnocytophagoides*	No	Yes	No
5	91/F	Intestinal perforation (intra-abdominal)	5.4, 5.9	*Escherichia coli*	*Bacteroides fragilis*	No	Yes	Yes
6	87/F	Cholangitis (intra-abdominal)	11.8, 12.3	*Enterobacter cloacae complex*, *Enterococcus faecium*, *Klebsiella pneumoniae*	*Prevotella buccae*, *Prevotella denticola*	No	Yes	No
7	61/M	Cholangitis, liver abscess (intra-abdominal)	6.6, 6.8	*Escherichia coli*, *Klebsiella oxytoca*	*Dysgonomonas capnocytophagoides*	No	Yes	No
8	59/M	Necrotizing soft tissue infection (skin and soft tissue)	3.2, 5.9	*Escherichia coli*, *Streptococcus constellatus*	*Eggerthella lenta*, *Lactobacillus brevis*, *Peptoniphilus* sp*.*, *Prevotella denticola*	No	Yes	Yes
9	76/F	Cholangitis (intra-abdominal)	9.5, 10.6^*a*^	*Klebsiella pneumoniae*	*Staphylococcus epidermidis*	Yes	No	No
10	76/F	Cholangitis (intra-abdominal)	3.7, 5.4	*Enterobacter cloacae* complex, *Enterococcus faecium*	*Finegoldia magna*	No	No	No
11	81/M	Non-occlusive mesenteric ischemia (intra-abdominal)	42.3^*b*^	*Bacteroides massiliensis*	*Cutibacterium acnes*	Yes	No	Not submitted
12	86/M	Cholangitis (intra-abdominal)	13.9^*b*^	*Enterococcus faecalis*, *Staphylococcus aureus*	*Cutibacterium acnes*	Yes	No	No
13	66/M	Intestinal perforation (intra-abdominal)	5.8, 5.9	*Klebsiella pneumoniae*	*Fusobacterium nucleatum*	No	Yes	Yes
14	78/F	Aspiration pneumonia (respiratory)	5.3, 11.2	*Serratia marcescens*, *Streptococcus intermedius*	*Mogibacterium timidum*	No	Yes	No
15	77/M	Surgical site infection after surgery for pharyngeal cancer (oropharynx)	11.0, 11.2	*Klebsiella pneumoniae*, *Streptococcus intermedius*, *Streptococcus mitis/oralis*	*Parvimonas micra*, *Solobacterium moorei*, *Veillonella parvula*	No	Yes	Yes^*c*^
16	84/F	Bacterial translocation from colorectal cancer (intra-abdominal)	22.1, 43.9^*d*^	*Clostridium ramosum*, *Parvimonas micra*	*Eggerthella lenta*	No	Yes	Not submitted
17	58/M	Intra-abdominal bleeding after liver transplantation (intra-abdominal)	10.6, 11.0	*Enterococcus faecium*, *Klebsiella pneumoniae*	*Veillonella parvula*	No	Yes	Not submitted
18	88/M	Diverticulitis (intra-abdominal)	18.7, 94.9^*e*^	*Streptococcus anginosus*	*Desulfovibrio fairfieldensis*	No	Yes	Not submitted
19	68/M	Cholangitis (intra-abdominal)	11.2, 12.8	*Enterococcus faecium*	*Veillonella parvula*	No	Yes	No
20	52/M	Diabetic foot infection (skin and soft tissue)	13.0, 15.9	*Providencia stuartii*, *Proteus mirabilis*, *Pseudomonas aeruginosa*, *Staphylococcus aureus*, *Streptococcus anginosus*	*Bacteroides fragilis*	No	Yes	Yes
21	61/F	Pyometra (female genital tract)	26.5, 26.5	*Peptoniphilus* sp.	*Campylobacter ureolyticus*	No	Yes	Not submitted
22	85/F	Unknown (others)	24.2, 27.2	*Streptococcus intermedius*	*Flavonifractor plautii*	No	No	Not submitted
23	81/F	Cholangitis (intra-abdominal)	9.3, 9.9	*Escherichia coli*, *Edwardsiella tarda*, *Vibrio fluvialis*	*Bacteroides fragilis*, *Fusobacterium ulcerans*	No	Yes	Yes
24	53/F	Pyelonephritis (urinary tract)	25.7, 28.6	*Bacteroides thetaiotaomicron*, *Corynebacterium striatum*	*Actinotignum schaalii*	No	Yes	Yes
25	78/M	Cholecystitis (intra-abdominal)	6.8, 6.8	*Escherichia coli*, *Klebsiella variicola*, *Enterococcus gallinarum*, *Clostridium perfringens*	*Pyramidobacter piscolens*	No	Yes	No
26	84/M	Intra-abdominal abscess (intra-abdominal)	24.2, 27.3	*Bacteroides pyogenes*, *Parvimonas micra*, *Streptococcus constellatus*	*Slackia exigua*	No	Yes	No
27	75/M	Necrotizing soft tissue infection (skin and soft tissue)	22.8, 30.6	*Parvimonas micra*, *Prevotella nanceiensis*, *Streptococcus anginosus*	*Actinomyces odontolyticus*	No	Yes	No
28	80/M	Diverticulitis (intra-abdominal)	8.8, 9.8	*Enterococcus faecium*, *Enterobacter cloacae complex*, *Bacillus subtilis*, *Morganella morganii*, *Clostridium perfringens*	*Eubacterium limosum*	No	Yes	No
29	70/F	Pyelonephritis (urinary tract)	11.3, 13.8	*Enterococcus faecalis*	*Actinotignum schaalii*	No	No	Yes

^*a*^ *Staphylococcus epidermidis* was detected only in the anaerobic bottle (TTP, 10.6 h) by the re-incubation method.

^*b*^ Only one anaerobic bottle was positive.

^*c*^ *Parvimonas micra* was the only microorganism isolated from the pus specimen obtained from the surgical site.

^*d*^ *Eggerthella lenta* was detected in the anaerobic bottle with a TTP of 43.9 h by the standard method.

^*e*^ *Desulfovibrio fairfieldensis* was detected in the anaerobic bottle with a TTP of 94.9 h by the standard method.

^*f*^ TTP, time to positivity. Values represent the time to positivity of each anaerobic bottle within the same blood culture set.

**TABLE 3:** Additional anaerobic organisms detected by the re-incubation method^*a*^

Microorganisms	*n*
Gram-negative bacilli	
*Bacteroides fragilis*	3
*Dysgonomonas capnocytophagoides*	2
*Prevotella denticola*	2
*Campylobacter concisus*	1
*Campylobacter ureolyticus*	1
*Desulfovibrio fairfieldensis*	1
*Dialister pneumosintes*	1
*Fusobacterium nucleatum*	1
*Fusobacterium ulcerans*	1
*Prevotella buccae*	1
*Pyramidobacter piscolens*	1
Gram-negative cocci	
*Veillonella parvula*	3
Gram-positive cocci	
*Parvimonas micra*	2
*Finegoldia magna*	1
*Peptoniphilus* sp.	1
Gram-positive non-spore-forming bacilli	
*Actinotignum schaalii*	2
*Eggerthella lenta*	2
*Actinomyces odontolyticus*	1
*Eubacterium limosum*	1
*Lactobacillus brevis*	1
*Mogibacterium timidum*	1
*Slackia exigua*	1
*Solobacterium moorei*	1
Gram-positive bacilli with variable sporulation	
*Flavonifractor plautii*	1

^*a*^ Two isolates of *Cutibacterium acnes* and one isolate of *Staphylococcus epidermidis* were regarded as contaminants and were therefore excluded from this summary.

## DISCUSSION

In this study, re-incubation of positive anaerobic blood culture bottles improved the detection of anaerobes compared with routine subculture, suggesting that some pathogens responsible for anaerobic bacteremia may be missed using conventional laboratory workflows. In the re-incubation-positive group, intra-abdominal infection was the most common source of bacteremia, followed by skin and soft tissue infections, and oropharyngeal infections. Although this finding may reflect the high proportion of intra-abdominal infections in our cohort, this result is consistent with previous reports identifying intra-abdominal infections as the most common source of anaerobic bacteremia (1–6).

Except for *B. fragilis* and *Fusobacterium* spp., most of the anaerobes detected only by the re-incubation method included species rarely reported to cause bacteremia. These organisms have been reported to exhibit prolonged TTP, typically 2–3 days for *Actinotignum schaalii* (12, 23), *Dialister pneumosintes* (13), *D. capnocytophagoides* (24–26), *E. lenta* (11, 27), *Flavonifractor plautii* (28), and *Slackia exigua* (29–31), and 3–6 days for *Desulfovibrio* spp. (15, 32). Although concordance between anaerobes detected only in the re-incubation-positive group and specimens from the infection focus was confirmed in only a few cases, the infection sources were generally consistent with those reported previously for the identified anaerobes.

*V. parvula* was among the microorganisms most frequently identified in the re-incubation-positive group (three cases), consistent with its known association with oral and intra-abdominal infections (33). The most common infection source for *A. schaalii* bacteremia is the urinary tract, particularly in older adults and those with underlying urological conditions, such as urinary obstruction, ureteral stents, and nephrostomy (12, 23, 34). In the two cases in which *A. schaalii* was identified by the re-incubation method, the infection focus was pyelonephritis due to ureteral stent obstruction, and *A. schaalii* was also detected in urine by anaerobic culture, consistent with previous reports. Oral and dental infections are the most common sources of *D. pneumosintes* bacteremia (13). *D. capnocytophagoides* has been reported in blood cultures from patients with cholangitis or liver abscess (24, 25). *Solobacterium moorei* and *P. micra* have been reported in patients with oropharyngeal infections (14, 35). The most common infectious focus of *E. lenta* bacteremia is intra-abdominal infection (11, 27). *Campylobacter ureolyticus* and *Campylobacter concisus* were identified among patients in the re-incubation-positive group. *C. ureolyticus* is the fourth most frequently detected species in *Campylobacter* bacteremia after *C. jejuni*, *C. coli*, and *C. fetus* (36, 37). *C. concisus* is typically isolated from oral or gastrointestinal specimens (38), and reports of bacteremia are rare (39). These findings suggest that bacteremia caused by slow-growing and infrequently reported anaerobic species may be missed in routine clinical practice.

Several studies have shown that next-generation sequencing (NGS) of positive blood culture specimens improves the detection of anaerobic bacteria compared with using conventional culture methods alone (10, 40). Hosoda et al. (10) suggested that the under-identification of anaerobic bacteria may be attributable to the difficulty in obtaining pure cultures and insufficient reference databases for matrix-assisted laser desorption/ionization time-of-flight mass spectrometry (MALDI-TOF MS). However, in this study, all organisms in the re-incubation-positive group except *D. fairfieldensis* were successfully identified using MALDI-TOF MS. This discrepancy may partly be explained by differences in the MALDI-TOF MS platforms used, as Hosoda et al. used the VITEK MS system (bioMérieux), whereas we used the MALDI Biotyper system (Bruker Daltonik GmbH), which use different proprietary reference databases to identify microorganisms. Although direct MALDI-TOF MS using Sepsityper or optimized in-house protocols can achieve species-level identification directly from positive blood cultures, its diagnostic performance remains limited in polymicrobial bloodstream infections (41, 42). In addition, the low bacterial load at the time blood cultures become positive may further compromise identification accuracy (43). Accordingly, although MALDI-TOF MS does not necessarily require colony formation, colony-based culture may still be important for complete identification in polymicrobial bacteremia, particularly when target organisms must be distinguished from coexisting microorganisms. For fastidious or slow-growing bacteria, cultivation may be difficult, and colonies may be overlooked or masked by those of other organisms. Conversely, culture-independent approaches, such as NGS, may enable the detection of a broader range of bacterial species and reduce culture-dependent bias. Nevertheless, slow-growing bacteria may not have proliferated sufficiently at the time of blood culture positivity, and their low bacterial burden may still limit the performance of direct detection methods.

In the re-incubation-positive group, two cases of *C. acnes* and one case of *S. epidermidis* were identified and considered contaminants. As *S. epidermidis* was not identified by the standard method, contamination during the initial subculture process was suspected. In contrast, given the reported TTP of 4–5 days for *C. acnes* (7), contamination at the time of blood culture collection could not be excluded.

Antimicrobial resistance among anaerobes is increasing, particularly to clindamycin, β-lactams, and carbapenems (44, 45). Among the additional organisms recovered by the re-incubation method, several species have been reported to exhibit clinically important antimicrobial resistance. *B. fragilis* demonstrates clindamycin resistance of 21%–49% and carbapenem resistance of 7%–13% (44, 45); *Prevotella* spp. show clindamycin resistance of 40%–53% (46); *Finegoldia magna* exhibits clindamycin resistance in up to 54% of isolates (46); and *E. lenta* has been reported to exhibit only 24.3% susceptibility to piperacillin-tazobactam (47). A significant limitation of this study is that antimicrobial susceptibility testing was not performed for the additional isolates recovered by the re-incubation method. Therefore, we could not determine whether these isolates harbored clinically relevant antimicrobial resistance that might have influenced empiric antimicrobial selection. Given the increasing prevalence of antimicrobial resistance among anaerobes and the emergence of multidrug-resistant strains, routine susceptibility testing is increasingly recommended for isolates recovered from sterile sites (48). Future studies incorporating antimicrobial susceptibility testing data would further clarify the clinical significance of these additional isolates and provide valuable information for optimizing empiric antimicrobial therapy and monitoring local antimicrobial resistance trends.

This study has several limitations. First, this was a single-center study conducted at a tertiary care hospital with a relatively small sample size, which may limit the generalizability of the findings. Second, we were unable to determine whether the anaerobes detected exclusively by the re-incubation method were concordant with culture results from the suspected infection focus in all cases, because specimens from the suspected infection focus were not available for all patients. Third, given the high frequency of polymicrobial bacteremia and the small colony size of anaerobes, the possibility of missed detection cannot be excluded. Fourth, because of the labor-intensive microbiological procedures required for this study, not all consecutive eligible cases during the study period were included, which may have introduced selection bias. Fifth, in this study, the duration of additional incubation of anaerobic blood culture bottles was set at 4 days; however, given the variability in TTP among bottles, the appropriateness of a uniform 4-day extension period was not evaluated. Sixth, our laboratory routinely incubated blood culture bottles for up to 7 days using the BacT/ALERT Virtuo system, whereas many laboratories use a 5-day incubation protocol in accordance with current Infectious Diseases Society of America and the American Society for Microbiology recommendations (49). However, all blood culture bottles included in this study became positive within 5 days, and the re-incubation method was initiated only after blood culture positivity. Therefore, the predefined maximum incubation period was unlikely to have affected the recovery of additional anaerobic organisms. Nevertheless, the applicability of our findings should be confirmed in laboratories using different blood culture protocols.

Seventh, we did not assess the clinical impact of identifying additional anaerobes recovered by the re-incubation method because these organisms were identified solely for research purposes and were not reported to the treating physicians. In addition, identification required approximately 8 days after blood culture positivity, by which time most patients had already shown clinical improvement. Consequently, we could not determine whether their identification would have influenced antimicrobial selection or patient outcomes. Moreover, many patients had infections, such as intra-abdominal or oropharyngeal infections, that had already been managed with appropriate source control, when indicated, and antimicrobial therapy with adequate anaerobic coverage before the additional organisms were identified. Therefore, the clinical significance of these additional organisms remains uncertain. Eighth, antimicrobial susceptibility testing was not performed for the additional anaerobic isolates recovered by the re-incubation method; therefore, their antimicrobial resistance profiles and potential influence on empiric antimicrobial therapy could not be evaluated. Finally, culture-independent molecular methods, such as next-generation sequencing, were not performed. Consequently, anaerobic organisms that were difficult to cultivate or obscured by coexisting microorganisms may have been missed, potentially leading to an underestimation of the true diversity of anaerobic organisms in polymicrobial bloodstream infections.

In conclusion, re-incubation of positive anaerobic blood culture bottles improved the detection of slow-growing anaerobes that may be overlooked by conventional subculture methods. Although routine implementation of this method is unlikely to be practical because of its relatively low additional detection rate and prolonged turnaround time, these findings provide valuable microbiological insights into the diversity of anaerobic bacteremia. Further studies using rapid molecular approaches are warranted to improve the detection of slow-growing anaerobes directly from positive blood culture specimens and to clarify their clinical significance.

## MATERIALS AND METHODS

### Study design and patient selection

Patients admitted to Hiroshima University Hospital between September 2023 and July 2025 were included in this observational study. The inclusion criteria were (i) age ≥18 years, (ii) collection of two sets of blood cultures, (iii) at least one anaerobic blood culture bottle positive, and (iv) blood culture positivity detected during weekday working hours. Because of the labor-intensive microbiological procedures required for this study, it was not feasible to include all consecutive eligible patients during the study period. If the same patient experienced multiple episodes of bacteremia during the study period, only the first episode was included in the analysis.

### Clinical data collection and definitions

Data were collected on the age and sex of the patient, source of bacteremia, and all microbiological results. An infection focus was defined by the presence of at least two of the following: (i) bacterial isolation from the suspected focus, (ii) radiological or laboratory findings suggestive of focal infection, and (iii) symptoms consistent with focal infection. Polymicrobial bacteremia was defined as the presence of two or more bacterial species in a single blood culture bottle or separate culture bottles from the same blood culture set. TTP was defined as the interval between the start of incubation of the blood culture bottle and the time at which the automated blood culture system signaled positive. For organisms recovered only after additional incubation of positive anaerobic blood culture bottles, true bacteremia or contamination was determined by infectious disease physicians based on the suspected infection focus, the patient’s clinical course, laboratory findings, imaging findings, the number of positive blood culture bottles, concordance with organisms recovered from the initial subculture, culture results from the suspected infection focus (when available), and the known pathogenic potential of the recovered organism.

### Microbiological analysis

Blood cultures were processed using the BacT/ALERT Virtuo system (bioMérieux). BacT/ALERT FA and FN Plus bottles (bioMérieux) were used. Approximately 8–10 mL of blood was inoculated into each blood culture bottle in accordance with the institutional protocol. As part of the routine institutional laboratory protocol, blood culture bottles were incubated for a maximum of 7 days. Positive anaerobic bottles were subcultured onto modified Drigalski agar plates (Eiken Chemical Co., Ltd., Tokyo, Japan) under aerobic conditions, 5% sheep blood agar plates, and chocolate agar plates (Kyokuto Pharmaceutical Industrial Co., Ltd., Tokyo, Japan) under 5% CO₂, and Brucella blood agar plates supplemented with hemin and vitamin K1 (Kyokuto Pharmaceutical Industrial Co., Ltd.) under anaerobic conditions using AnaeroPack (Mitsubishi Gas Chemical Co., Inc., Tokyo, Japan) at 36°C. Anaerobic cultures were incubated for 4 days during the study period, and this procedure was designated as the “standard method.”

**Fig 1:**
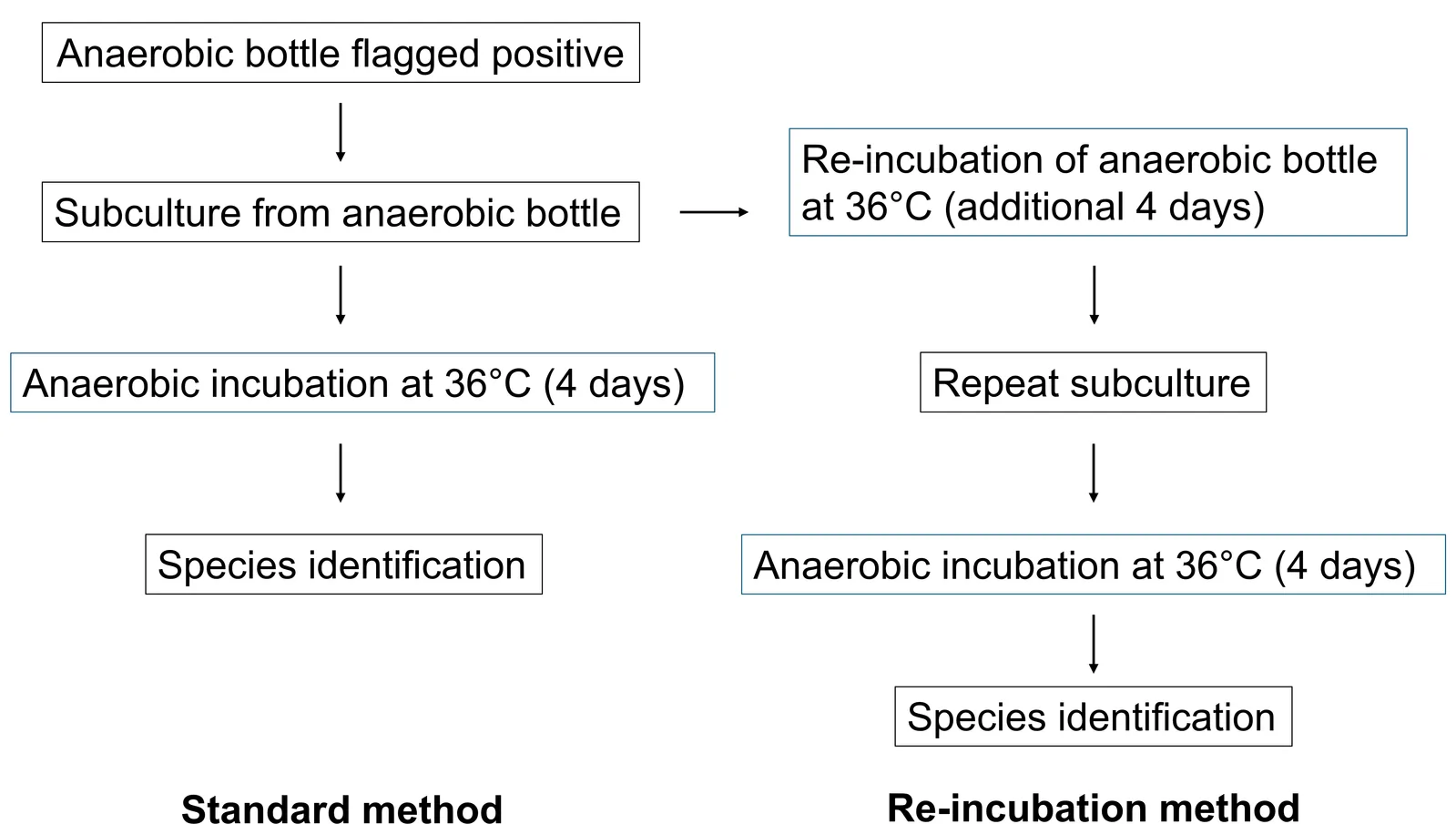
Workflow of the standard and re-incubation methods for processing positive anaerobic blood culture bottles.

After an anaerobic blood culture bottle signaled positive and the initial subculture had been performed, the bottle was re-incubated at 36°C for an additional 4 days. This re-incubation period was separate from the routine 7-day blood culture incubation protocol. Subsequently, repeat subculture was performed onto Brucella blood agar supplemented with hemin and vitamin K1 (Kyokuto Pharmaceutical Industrial Co., Ltd.), followed by incubation under anaerobic conditions at 36°C for a further 4 days. If mixed growth was observed on the subculture plates, additional subculture was performed as necessary to obtain pure isolates for identification. Identification of additional anaerobic organisms required at least 8 days after the initial positive blood culture signal and occasionally longer when additional subculture was required to isolate slow-growing anaerobes from mixed cultures. This procedure was designated as the “re-incubation method” (Fig. 1). A 4-day re-incubation period was selected to balance the detection of slow-growing anaerobes with the practical constraints of routine clinical laboratory workflows based on previous reports that a 4–5-day incubation period is sufficient for detecting most bloodstream pathogens using contemporary automated systems (7, 49, 50).

Bacterial species were identified by MALDI-TOF MS using a MALDI Biotyper Sirius system (Bruker Daltonik GmbH, Bremen, Germany) and MBT Compass 4.1 software with the MBT Compass library (Version 10, 9607MSPs). Identification was initially attempted using the direct transfer method according to the manufacturer’s instructions. If reliable species-level identification could not be achieved, on-plate formic acid extraction was performed. Ethanol/formic acid tube extraction was subsequently performed only when reliable identification remained unsuccessful. In accordance with the manufacturer’s criteria, identification scores  ≥2.0 were considered reliable for species-level identification. Isolates that could not be identified using MALDI-TOF MS were subjected to 16S rRNA gene sequencing for species-level identification as previously described (12). For the analysis, patients in whom at least one anaerobic bottle yielded additional anaerobic bacteria detected by the re-incubation method but not by the standard method were assigned to the re-incubation-positive group, and all the other patients were assigned to the standard-method-only group. When available, specimens from the suspected infection focus were cultured using the standard methods described above.

### Statistical analysis

Categorical variables were reported as frequencies and percentages, whereas continuous variables were reported as medians and interquartile ranges. Categorical variables were compared between groups using the chi-square test or Fisher’s exact test, and continuous variables were compared between groups using the Mann–Whitney U test. Two-sided *P* values of <0.05 were considered statistically significant. Statistical analyses were performed using JMP Pro software (version 18.0; SAS Institute Inc., Cary, NC, USA).

## Data Availability

The data supporting the findings of this study are available from the corresponding author on reasonable request.
